# Frequency of breakfast consumption is inversely associated with the risk of depressive symptoms among Chinese university students: A cross-sectional study

**DOI:** 10.1371/journal.pone.0222014

**Published:** 2019-08-30

**Authors:** Zhihong Zhu, Yufei Cui, Qiang Gong, Cong Huang, Feng Guo, Wang Li, Wenbo Zhang, Yanbo Chen, Xin Cheng, Yongxiang Wang

**Affiliations:** 1 Institute of Exercise Epidemiology and Department of Physical Education, Huaiyin Institute of Technology, Huaian, People’s Republic of China; 2 Department of Physical Education, Zhejiang University, Hangzhou, People’s Republic of China; 3 Department of Physical Education, Changchun Institute of Education, Changchun, People’s Republic of China; 4 Department of Physical Education, Dalian Institute of Science and Technology, Dalian, People’s Republic of China; Gazi University, Faculty of Health Sciences, TURKEY

## Abstract

**Introduction:**

Eating breakfast has been proved to positively influence human health. However, evidence for the association between breakfast consumption and depressive symptoms is lacking, especially among young adults. The aim of this study was to determine whether breakfast consumption is associated with depressive symptoms among Chinese university students.

**Methods:**

A cross-sectional study involving 10,174 undergraduate students (6,287 males and 3,887 females) was conducted in 2015. Breakfast consumption was assessed using a self-reported questionnaire. Depressive symptoms were assessed using the Zung self-rating depression scale (SDS) score. Three cut-off values of the SDS score (40, 45, and 50) were used to assess the severity of the depressive symptoms. Logistic regression models were used to analyze the association between the frequency of breakfast consumption and depressive symptoms.

**Results:**

The mean SDS score was 37.1±8.0 in present study. In crude model, a higher frequency of breakfast consumption was primarily associated with a lower prevalence of depressive symptoms in all three SDS groups (p = 0.001, < 0.001, = 0.009 for SDS cut-off value 40, 45, and 50 points, respectively). These associations remained significant after adjustment for confounding factors.

**Conclusions:**

A higher frequency of breakfast consumption was strongly associated with a lower prevalence of depressive symptoms among Chinese university students. These results corroborate the evidence that the habit of eating breakfast may be beneficial to mental health.

## Introduction

The prevalence of depressive symptom has dramatically increased over the past decades [1]. Depression is one of the most prevalent mental disorders worldwide [2], and is associated with adverse health outcomes, such as functional impairment [3, 4], cardiovascular disease [5], and mortality among older adults [6]. In addition depression is a major mental health issue among young adults, and a risk factor on health status [7]. Recent reports involving university students have indicated that depressive symptoms influenced their life quality, academic productivity, physical function, and relationship with family [8, 9]. Depressive symptoms have also been shown to be a risk factor for suicide attempts among college students [10, 11]. Moreover, a higher risk of developing depressive symptoms has been reported in college students than in the general population [12]. As young adulthood is an important period in the transition from adolescence to adulthood, it is crucial to maintain a healthy mental status in this period.

Meanwhile, breakfast is the first and most important meal of the day, providing 20–35% of the total daily energy needs [13]. Breakfast consumption has been associated with a good diet quality, better nutrient intake, and better physical fitness [14, 15]. Conversely, skipping breakfast is often associated with adverse outcomes, such as weight gain [16], and obesity [17, 18]. In addition, fiber and calcium intake have been shown to be significantly higher in people who eat breakfast [19, 20]. As obesity is a risk factor for depressive symptoms [21], and a higher fiber and calcium intake are associated with a lower prevalence of depressive symptoms [22, 23], one might say that breakfast consumption is associated with depressive symptoms. However, studies investigating such associations are scarce. Most studies have focused on adults (20 years or older) or seniors (65 years or older) [24, 25], and the evidence supporting this association in adolescents and young adults is limited. Thus, we designed a cross-sectional study to investigate the association between breakfast consumption and depressive symptoms in Chinese university students.

## Materials and methods

### Participants

This cross-sectional study was conducted at the Dalian Institute of Science and Technology (Liaoning province, China). Physical health monitoring was performed every year from 2007. All students who underwent physical examination were invited to participate in the study in 2015. Self-reported questionnaires were obtained from students who agreed to participate. (N = 10,570). Individuals with missing data in the self-reported questionnaire (N = 396) were excluded. The final sample comprised 10,174 subjects (6,287 males and 3,887 females).

### Ethics statement

This study was approved by Human Investigation Ethics Committee of Dalian Institute of Science and Technology. Students who agreed to participate provided written informed consent.

### Depressive symptoms

The severity of the depressive symptoms were measured using the Chinese version of the Zung Self-Rating Depression Scale (SDS) [26]. Previous studies have demonstrated its reliability and validity in Chinese populations [27]. SDS is a 20-item, self-reported assessment in which participants indicate on a four-point scale, either positive or negative. The overall score is a sum of the scales corresponding to the responses to the 20 items and its value ranges from 20 to 80 points. In the present study, we used three cut-off values (40, 45, and 50 points) to assess the severity of the depressive symptoms, with greater values indicating increased severity [28].

### Breakfast consumption

Breakfast consumption was measured using a standard questionnaire which has been used in a previous study [15]. Responses to the following question determined the frequency of breakfast consumption: “How many days did you have breakfast per week in previous month?” The answers were “never,” “less than once,” and ranging from “once” to “7 times.” We classified the answers into three categories: 6–7 times (consumers), 2–5 times (occasional consumers), and less than 2 times (skippers).

### Confounding factors

Body weight and height were measured before physical examinations and expressed as kilograms and centimeters, respectively. Body mass index (BMI) was calculated from the weight (kg) divided by the height (m) squared. Physical activity intensity (classified as low, moderate or vigorous) was assessed using the short form of the International Physical Activity Questionnaire (IPAQ), and the total metabolic equivalent of task (MET) was used to assess the energy cost of physical activities (MET-minutes/week). The response to the IPAQ form was used as the continuous variable. Information on age, sex, grade, living status, sleep quality, smoking and drinking status was obtained from a questionnaire survey. Tobacco smoking and alcohol drinking were assessed by two questions: “Are you a current smoker?” (Answer options: “yes” and “no”) and “How often do you drink?” (Answer options: “every day”, “occasionally” and “non-drinker”).

### Statistical analysis

Statistical analyses were performed using the SPSS software version 17.0 (SPSS, Inc., Chicago, IL). Depressive symptoms were used as the dependent variable, and frequency of breakfast consumption was used as the independent variable. Difference among categories of breakfast consumption were assessed by analysis of variance (for continuous variables) and logistic regression analysis (for proportional variables). Multiple logistic regression analysis was performed to examine the association between breakfast consumption and depressive symptoms in Model 1 (adjusted for sex, grade, BMI, and ethnicity) and Model 2 (adjusted for Model 1 in addition to physical activity, living status, sleep quality, smoking and drinking). A p value below 0.05 indicated statistical significance.

## Results

Data were obtained from 10,174 students, of whom 6,287 were male (61.8%) and 3,887 were female (38.2%). The mean age ± standard deviation (SD) was 19.76±0.86 years. The general characteristics of the participants according to breakfast consumption are shown in **Table 1**. The frequency of breakfast consumption was inversely associated with BMI (p < 0.001) and SDS score (p = 0.001). The frequencies of the answers “every day” (p = 0.014) and “occasionally” (p = 0.003) (regarding alcohol drinking) and of individuals reporting good sleep quality (p < 0.001) were significantly higher in the “breakfast consumer” group (6–7 times/week), which also had the lowest rates of non-alcohol-drinkers (p < 0.001) and individuals reporting normal and poor sleep quality (p < 0.001 for both).

**Table 1:** Characteristics of participants according to breakfast consumption.

	Breakfast consumption			p for trend^a^
	Skipper	Occasional consumer	Consumer	
N	2798	2941	4435	
Sex (man; %)	61.3	63.2	62.1	0.738
BMI (kg/m^2^)^b^	22.8 (22.6, 22.9)^c^	22.5 (22.3, 22.6)	22.1 (22.0, 22.2)	< 0.001
Grade (%)				
First year	31.2	29.7	29.4	0.112
Second year	29.2	27.6	28.5	0.645
Third year	24.1	25.8	25.9	0.109
Fourth year	15.5	16.8	16.2	0.519
Minority ethnicity (%)	6.5	6.7	7.3	0.195
Living status (dormitory; %)	91.4	92	91.9	0.449
Physical activity (≥23METs h/week; %)	38.2	35	37.6	0.883
Smoking status (%)				
Smoker	13.1	13.6	12.7	0.767
Drinking status (%)				
Drinking everyday	4.5	5.6	5.9	0.013
Drink occasionally	25.1	24.1	27.9	0.003
Non-drinker	70.4	70.3	66.2	< 0.001
Sleep quality (%)				
Good	19.8	27.1	32.3	< 0.001
Normal	58.6	57.0	50.0	< 0.001
Poor	21.6	15.9	17.7	< 0.001
SDS score	37.5 (37.2, 37.8)	37.2 (36.9, 37.5)	36.9 (36.6, 37.1)	0.001

^a^ Obtained by using ANOVA for continuous variables and logistic regression analysis for variables of proportion.

^b^ BMI: body mass index; SDS: Self-rating depression scale.

^c^ Mean; 95% CI in parentheses (all such values).

**Table 2**shows the adjusted associations between frequency of breakfast consumption and depressive symptoms in subjects classified according to the SDS score. The prevalence of depressive symptoms was 35.9% in individuals with SDS > 40, 19.4% in individuals with SDS > 45, and 7.8% in individuals with SDS > 50. In the crude model, multiple logistic regression analyses showed an inverse association between breakfast consumption and risk of depressive symptoms in all SDS groups (p = 0.001 for SDS > 40, p < 0.001 for SDS > 45, and p = 0.009 for SDS > 50). Considering that some factors may influence the association between breakfast consumption and depressive symptoms, the variables in the Model 1 were adjusted for sex, grade, ethnicity and BMI. In the Model 2, the variables were additionally adjusted for drinking and smoking habits, physical activity, living status and sleep quality. However, the association between breakfast consumption and depressive symptoms remained significant in all SDS groups.

**Table 2:** Adjusted associations between frequency of breakfast consumption and depressive symptoms.

	Breakfast consumption			p for trend^a^
	Skipper	Occasional consumer	Consumer	
All subjects				
N	2798	2941	4435	
Depressive symptoms SDS ≥ 40				
N	1055	1086	1507	
Crude	1.00	0.97 (0.87, 1.08)^b^	0.85 (0.77, 0.94)	0.001
Model 1^c^	1.00	0.98 (0.88, 1.09)	0.87 (0.79, 0.96)	0.003
Model 2^d^	1.00	1.01 (0.90, 1.12)	0.90 (0.81, 1.00)	0.027
Depressive symptoms SDS ≥ 45				
N	608	574	787	
Crude	1.00	0.87 (0.77, 0.99)	0.78 (0.69, 0.88)	< 0.001
Model 1^c^	1.00	0.88 (0.78, 1.00)	0.79 (0.70, 0.89)	< 0.001
Model 2^d^	1.00	0.90 (0.79, 1.02)	0.81 (0.72, 0.91)	0.001
Depressive symptoms SDS ≥ 50				
N	249	224	318	
Crude	1.00	0.84 (0.70, 1.02)	0.79 (0.67, 0.94)	0.009
Model 1^c^	1.00	0.86 (0.71, 1.04)	0.81 (0.68, 0.96)	0.020
Model 2^d^	1.00	0.88 (0.72, 1.06)	0.82 (0.69, 0.98)	0.033

^a^ Obtained by multiple logistic regression analysis.

^b^ Adjusted OR; 95% CI in parentheses (all such values).

^c^ Adjusted for sex, grade, ethnicity and body mass index.

^d^ Adjusted for Model 1 plus drinking and smoking habits, physical activity, living status and sleep quality.

## Discussion

This study investigated the relationship between breakfast consumption and depressive symptoms in Chinese young adults. We found that breakfast skipping was associated with increased risk of depressive symptoms and these associations were not changed after adjustment for a number of potentially confounding variables. The mean SDS score of previous studies on Chinese university students were most more than 40 [29, 30]. We found that the mean SDS score in our participants was 37.1 (SD 8.0), which was lower than those studies. Moreover, the prevalence of depressive symptoms in individuals with SDS ≥ 50 was 7.8%, which also lower than that reported in previous studies [31, 32].

Research on eating behaviors and mental health is a topic of worldwide concern. Most studies have shown that healthy eating behaviors can have a beneficial effect on mental health. However, to our knowledge, studies investigating such associations among the Chinese population are scarce. Our findings are consistent with those of a Japanese study in 2019 that involved 716 employees aged 19 to 68 and those of a Korean study in 2017 that involved subjects aged 20 to more than 80 years. Both studies showed a higher likelihood of depressive behavior in subjects who consumed breakfast rarely or occasionally than in those who did it frequently [24, 33]. In addition, a Japanese study reported a significant association between irregular breakfast consumption and a high prevalence of depression in male white-collar workers [34]. A cross-sectional study in 2019 revealed that the greater the frequency of skipping breakfast, the greater the probability of experiencing depressive symptoms [35]. A short communication in 2003 confirmed that young adults who regularly consume breakfast report better health, including mental health [36]. Furthermore, a study examining the associations between lifestyle factors and depressive symptoms in college students found an inverse association between frequency of breakfast consumption and Center for Epidemiologic Studies Depression Scale (CES-D) score [37]. This result is partially consistent with that of our study. However, cut-off point of CES-D was not used in that study to define depressive symptom. In our study, we used three cut-off values of the SDS to define the levels of depressive symptoms. Based on these previous studies, our present finding strengthened the evidence on the relationship between breakfast consumption and mental health.

As aforementioned, this association can be explained by the effect of body weight, as body weight is positively associated with the prevalence of depression [21, 38]. We found a significant association between low frequency of breakfast consumption and higher body weight, which supports the results of a previous study [16]. However, when adjusted for BMI, the associations remained significant, that is, breakfast consumption was associated with depressive symptoms exists irrespective of BMI in present study. Previous study explain this association by the effect of carbohydrate ingestion. They indicated when level of blood glucose decrease at night time fast, cortisol are released. High level of cortisol is associated with increased inflammatory cytokine which especially attenuates serotonin, which alleviates depression [24]. Otherwise, breakfast consumption may modulate metabolic responses on fasting condition to maintain a supply of nutrients to the central nervous system, or effects on nutrient intake and status [39]. Nutrition is essential for the functioning of the central nervous system, including the regulation of neurotransmitters such as serotonin and dopamine, which can play a key role in the association between nutrition and depressive symptoms [40]. Thus, breakfast consumption may improve or prevent depressive symptoms.

The strengths of our study include the large sample size, and the analyses of various confounding factors. In addition, we used three cut-off values to categorize the individuals according to their SDS score. However, some methodological limitations must also be cited. First, selection bias must also be taken into account. In present study, 10,174 university students in Liaoning province participated. According to the government, the number of university student was about 647,000 in Liaoning province 2016. The participation rate was low, and our subjects were probably not representative of all Chinese university students in the general population. Second, due to the cross-sectional nature, it is difficult to draw conclusions about causality. Third, the data used in this population-based study were collected retrospectively, using self-report questionnaires, increasing the likelihood of recall bias. Fourth, although several confounding factors were adjusted for analysis, we could not rule out residual confounding.

In conclusion, the results of the present study provide evidence of association between frequency of breakfast consumption and depressive symptoms. These findings underline the significance of skipping breakfast as a risk factor for depressive symptoms among young adults and suggest that eating breakfast is crucial for reducing the incidence of depressive symptoms among university students. Further interventional studies are required to confirm these findings and better determine the casual link between these variables.
